# Crucial markers showing the risk of coronary artery disease in obesity: ADMA and neopterin

**DOI:** 10.5937/jomb0-24683

**Published:** 2020-10-02

**Authors:** Emre Avci, Alpaslan Karabulut, Avci Gulcin Alp, Burcu Baba, Cumhur Bilgi

**Affiliations:** 1 Hitit University, Faculty of Science and Arts, Department of Molecular Biology and Genetics, Corum, Turkey; 2 Hitit University, Faculty of Medicine, Department of Internal Medicine, Corum, Turkey; 3 High Education University, Faculty of Medicine, Department of Biochemistry, Ankara, Turkey

**Keywords:** obesity, asymmetric dimethylarginine, neopterin, endothelial dysfunction, endotelna disfunkcija, neopterin, asimetrični dimetilarginin, gojaznost

## Abstract

**Background:**

Obesity is responsible for high morbidity and mortality, both in developed and developing countries. It is associated with many chronic and metabolic diseases. Asymmetric dimethylarginine (ADMA) has been demonstrated to be a biomarker of endothelial dysfunction in humans and increased ADMA associated with cardiovascular disease (CVD) risk has been reported in many states. Neopterin (NP) produced by monocytes/macrophages in response to stimulation by interferon-gamma (IFN-γ) is emphasized in recent findings. The current study aims to investigate ADMA and NP levels which may assume a role in guiding the early diagnosis of coronary artery disease in obesity.

**Methods:**

This is an original research study in which ADMA and NP levels of 50 patients (25 male/25 female) diagnosed with obesity were compared with those of 30 healthy individuals (15 male/15 female) as control. The high-performance liquid chromatography (HPLC) method was used while determining parameters.

**Results:**

ADMA and NP levels in obese individuals were found to be significantly higher than in those enrolled in the control. ADMA values were found to be higher in obese subjects (0.71±0.24 μmol/L) as compared with levels found in healthy subjects (0.58±0.16 μmol/L) (p<0.05). A significant increase of serum neopterin levels was found in obese subjects (8.8±3.5 μmol/L) as compared with controls (4.9±1.69 μmol/L) (p<0.05). Also, there was a strong positive correlation between NP and ADMA values in obese individuals (r=0.954).

**Conclusions:**

Our study revealed that obese subjects have higher ADMA and neopterin levels. These results demonstrated that both ADMA and NP levels may be potential risk factors for coronary heart disease in obesity.

## Abstract (sr)

**Uvod:**

Gojaznost je odgovorna je za visoki morbiditet i mortalitet, kako u razvijenim zemljama tako i u zemljama u razvoju. Povezana je sa mnogim hroničnim bolestima i bolestima metabolizma. Dokazano je da je asimetrični dimetilaginin (ADMA) biomarker endotelne disfunkcije kod ljudi, a u mnogim državama je uočeno da je povećan ADMA povezan sa rizikom od kardiovaskularnih bolesti (CVD) prijavljen je u mnogim državama. U mnogim istraživanjima skorijeg datuma ukazano je na neopterin (NP) proizveden monocitima/makrofazima kao odgovor na stimulaciju interferon-gama (IFN-γ). Cilj ove studije je da se ispita nivo ADMA i NP koji mogu biti smernica za ranu dijagnoze bolesti koronarnih arterija kod gojaznih pacijenata.

**Metode:**

Ovo je originalna istraživačka studija u kojoj su upoređeni nivoi ADMA i NP kod 50 pacijenata (25 muškaraca/25 žena) kojima je dijagnostikovana gojaznost sa istim vrednostima kod 30 zdravih pojedinaca (kontrolna grupa od 15 muškaraca/15 žena). Pri određivanju parametara korišćena je metoda tečne hromatografije visokih performansi (HPLC).

**Rezultati:**

Utvrđeno je da su nivoi ADMA i NP kod gojaznih pojedinaca značajno viši od onih utvrđenih kod pojedinaca iz kontrolne grupe. Otkriveno je da su vrednosti ADMA veće kod gojaznih ispitanika (0,71 ± 0,24 mmol/L) u poređenju sa vrednostima kod zdravih ispitanika (0,58 ± 0,16 mmol/L) (p < 0,05). Utvrđeno je značajno povećanje nivoa neopterina u serumu kod gojaznih ispitanika (8,8 ± 3,5 mmol/L) u poređenju sa kontrolnom grupom (4,9 ± 1,69 mmol/L) (p < 0,05). Takođe, utvrđena je visoka pozitivna korelacija između vrednosti NP i ADMA kod gojaznih pojedinaca (r = 0,954).

**Zaključak:**

Naše istraživanje je otkrilo da gojazni ispitanici imaju viši nivo ADMA i neopterina. Ovi rezultati su pokazali da i ADMA i NP nivoi mogu biti potencijalni faktori rizika za koronarnu bolest srca kod gojaznih pacijenata.

## Introduction

Obesity is a growing worldwide health concern affecting more than half of the global adult population according to the World Health Organization (WHO) [1]. Obesity prevalence is rising in developed countries, as well as in developing countries [2] [3]. Many factors such as lifestyle factors, epigenetics, and genetics, have been associated with obesity pathogenesis. Obesity is related to enhanced risks of adverse health outcomes [4]. It is a heterogeneous state characterized by excess adiposity, and it increases the risk of development of metabolic and cardiovascular diseases (CVD) [5]. Insulin resistance (IR) has been proposed as a link between obesity and CVD [6]. Excessive ectopic lipid accumulation causes insulin resistance and local inflammation. In fact, overnutrition stimulates uncontrolled inflammatory responses in white adipose tissue, resulting in chronic low-grade inflammation, thus promoting the IR progression [4]. Obesity-associated disorders including hypertension, dyslipidemia, and diabetes mellitus lead to atherosclerosis prone milieu and, therefore, the development of CVD [1].

Endothelial dysfunction is considered as a stage of atherosclerosis and an important prognostic marker for CVD that contains several functional alterations in the vascular endothelium, such as impaired vasodilation, angiogenesis, increased of endothelial products and inflammatory activation [7] [6]. It is present in obesity and associated comorbidities. However, mechanisms associated with endothelial dysfunction and obesity have not yet been fully elucidated [6]. Endothelial dysfunction is well-known to precede coronary artery disease (CAD). Several cardiovascular risk factors, systemic or local inflammation and metabolic diseases elicit endothelial dysfunction. Nitric oxide (NO) is an endothelium-derived vasoactive molecule that plays a crucial role in sustaining endothelial homeostasis [8]. Endothelial dysfunction is related to reduced nitric oxide (NO) bioavailability which may be attributed to an increase in asymmetric dimethylarginine (ADMA) levels [8] [9]. ADMA has been demonstrated to be a biomarker of endothelial dysfunction in humans and increased ADMA levels have been reported in many studies associated with a high CVD risk [9] [10].

The functional and structural adaptations of the cardiovascular system due to obesity lead to the prothrombotic and proinflammatory environment [1]. Inflammation plays a vital role in atherosclerosis [11]. A significant role of macrophages in early atherogenesis is emphasized by recent findings which state that neopterin (NP) which is produced by monocytes/macro phages in response to stimulation by interferongamma (IFN-γ) is an important determinant of outcome in subjects with angiographic CAD [12] [13]. It was figured out that neopterin levels are elevated in diabetes, cardiovascular diseases, and obesity [11] [14].

In this study, we aimed to evaluate ADMA and neopterin levels in obese and healthy subjects and to investigate their relationship with each other and with clinical and metabolic parameters and cardiovascular risk factors.

## Materials and Methods

### Patients

Individuals who were admitted to the Internal Diseases Clinic and who were diagnosed with obesity were included in this study. Our study was approved by the Local Ethics Committee. A power analysis was used to determine sample size before the study. According to the power analysis, 50 obese individuals (25 female/25 male) and 30 healthy individuals (15 female/15 male) were included in our study. Subjects were classified according to body mass index (BMI). The patient group comprised of 50 obese individuals with BMI≥30 (35.9±5.4 kg/m^2^) with a mean age of 41.0±6.1 years, while the control group comprised of 30 healthy individuals with BMI<30 (20.9±3.5 kg/m^2^) with a mean age of 39.6±3.9 years. Samples of these healthy individuals constituted the control group of the study.

Blood samples were centrifuged at 4000 g for 5min. at 4 °C and were stored at -80 °C until the analysis. Before samples were stored, routine biochemical parameters were evaluated via an autoanalyzer (Roche Cobas® 6000 C501, Roche Diagnostic Germany) in all samples (Table 1). Fasting plasma glucose was measured by the enzymatic colorimetric method using glucose oxidize test (intra-and interassay coefficients of variation 2.4% and 2.9%, respectively). Insulin was measured by radioimmunoassay (Immunotech, Prague, Czech Republic). Sensitivity was 0.5 μU/mL, and the upper limits of intra-and inter-assay coefficients of variation were 4.1 and 3.8, respectively. HOMA-IR was used to evaluate insulin resistance (fasting serum insulin (μU/mL) × fasting plasma glucose (mmol/L l-1)/405) (Esteghamati, 2010). Homo-IR Cut-off values were 2.47 (sensitivity, 0.44; specificity, 0.74).

**Table 1:** Demographics and some biochemical data of healthy and obese individuals (mean±SD, min.-max.) S.D: Std. Deviation, SEM: Std. Error of Mean. The statistical significance between the groups was evaluated with the Man-Whitney U test, and only the age parameter did not showany statistical significance between the groups (*: p>0.05). There was a significant intergroup difference in the other parameters(p<0.05) is not statistically significant.

	Controls (n=30)			Obese Individuals (n=50)		
	Mean±S.D	SEM	Min-Max	Mean±S.D	SEM	Min-Max
Age (years)*	39.6±3.9	0.71	33.0–51.0	41.0±6.1	0.86	32.0–56.0
Weight (kg)	64.0±4.5	0.81	57.0–78.0	107.0±13.2	1.86	93.0–155.0
Body mass index (kg/m^2^)	20.9±3.5	0.63	16.0–28.0	35.9±5.4	0.76	19.0–48.0
Fasting Blood Glucose (mmol/L)	88.3±5.8	1.06	78.0–96.0	109.1±13.8	1.95	93.0–147.0
Insulin (μU/mL)	9.11±3.1	0.56	4.0–18.0	16.5±5.4	0.76	8.0–29.0
HOMA-IR	1.98±0.64	0.11	0.84–3.73	4.48±1.62	0.23	2.0–8.7
Total Cholesterol (mmol/L)	3.75±0.57	0.10	2.48–4.65	4.64±1.72	0.24	130.0–440.0
LDL-Cholesterol (mmol/L)	2.46±0.43	0.07	2.07–3.62	3.11±0.86	0.12	96.0–199.0
HDL-Cholesterol (mmol/L)	0.57±0.08	0.01	0.41–0.72	1.00±0.12	0.18	33.0–48.0
Triglycerides (mmol/L)	1.49±0.08	0.01	1.37–1.75	2.22±0.29	0.41	136.0–246.0
Prevalence of risk factors		Yes/No	Positivity (%)	Yes/No		Positivity (%)
Hypertension		1/29	32.0	16/34		3.4
Diabetes mellitus		2/28	30.0	15/35		6.6
Cardiovascular diseases		0/30	22.0	11/39		0.0
Smoking		0/30	86	7/43		0.0

### Measurement of ADMA

The asymmetric dimethylarginine (ADMA) as anendothelial damage marker was performed using the modified method by Avci et al. (2008) [15]. The working conditions and system parameters of the ADMA method are shown in Table 2.

**Table 2:** The working conditions and system parameters of the HPLC system used in the study

Working condition	System Parameters	
	ADMA	Neopterin
HPLC Model	Agilent Technologies 1200 Series	
Sensor	Flouresans Sensor	
Mobile Phase	6.8 pH 50 millimolarsodium acetate buffer	6.4 pH 0.015 mol/L phosphate buffer
Column materialAnalytical ColumnProtector Column	4.6 x 150 mm, Phenomenex Hypersil ODS-2, C_18_ 5 μm column Phenomenex ODS-2, C_18_ 5 μm Phase Cartridge	4.6 x 250 mm, Allsphere ODS-2, C_18_ 5 μm reverse-phase Spherisorb ODS-2, C_18_ 5 μm reverse-phase
Wavelength	338 nm excitation and 425 nm emission	353 nm excitation and 438 nm emission
Flow Rate	1.0 mL/minute	0.8 mL/minute
Sample Volume	10 μL	20 μL
Operation time	35 minute	20 minute
Temperature	37 °C	30 °C
Discrimination	* Reverse phase ion exchange *	

### Measurement of Neopterin

**Figure 1:**
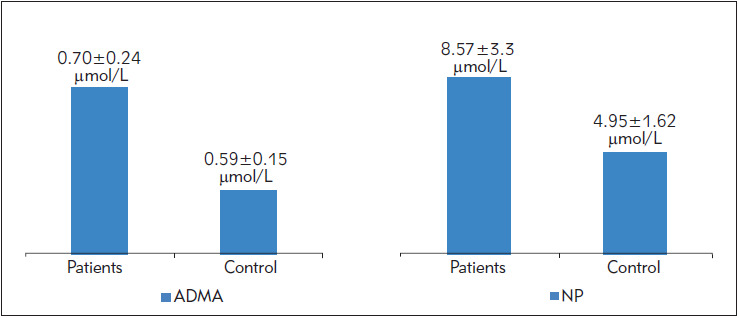
The ADMA and neopterin levels in both patients with obese and control subjects.

The measurement of neopterin (NP) was carried out by HPLC using a method by Avci et al. (2008) [15]. The working conditions and system parameters of the NP method are shown in Table 2. (Figure 1).

### Statistical Analysis

The findings of the study were evaluated statistically using IBM SPSS Statistic 22.0 (IBM Co., Armonk, NY, USA). All data were given as mean± standard deviation. The statistical significance level was defined as 0.05. Shapiro-Wilk test was used to check whether the data were suitable for normal distribution. Mann Whitney-U test was preferred for nonparametric tests due to a lack of normal distribution of data. The correlations among parameters were observed by Spearman's rho correlation test.

## Results

The demographic data and some biochemical parameters of patients and healthy groups were summarised in Table 1. The suitability of the data for normal distribution was examined by the Shapiro-Wilk Test. It was determined that the parameters did not have a normal distribution (0.000<p<0.005). Therefore, non-parametric tests were used for statistical analysis of the parameters. According to the nonparametric Mann-Whitney U test, there was no statistically significant difference between the groups in the age parameter. When the other demographic data and biochemical parameters were examined, statistically significant differences were found between the groups. These demographic and biochemical parameters were found to be significantly higher in obese individuals compared to those in the controls (p<0.05).

When examined, the correlations among parameters were encountered among parameters. Positive correlations were found among weight and BMI, Fasting Blood Glucose, Insulin, HOMA-IR, Total Cholesterol, LDL-Cholesterol, and Triglyceride (0.453<r<0.740). Also, other positive correlations were found among BMI and Fasting Blood Glucose, Insulin, HOMA-IR, Total-Cholesterol, LDL-Cholesterol and Triglyceride (0.395<r<0.725). Additionally, positive correlations were also found among HOMA/IR and Fasting Blood Glucose, Insulin, HOMA-IR, Total Cholesterol, LDL-Cholesterol and Triglyceride (0.365<r<0.964).

ADMA and NP parameters were not suitable for normal distribution according to the Shapiro-Wilk test. For this reason, the non-parametric Mann Whitney U test was used. According to this test, both ADMA and NP parameters were found to be higher in the patient group compared to the control group. The increased ADMA values were found in obese subjects (0.70±0.24 μmol/L, SEM: 0.43, Min.-Max: 0.43-1.16) compared to the levels found in healthy subjects (0.59±0.15 μmol/L, SEM: 0.02, Min-Max: 0.38-0.82). ADMA levels were significantly higher in obese subjects (U: 548.000, p=0.045, p<0.05).

The data obtained from all the participants in the study showed that neopterin levels were variable in obese individuals. However, a significant increase of serum neopterin levels was found in obese subjects (8.57±3.3 mmol/L, SEM: 0.60, Min-Max: 4.8-18.4) as compared with controls (4.95±1.62 μmol/L, SEM: 0.22, Min-Max: 2.6-9.4). NP levels were significantly higher in obese subjects (U: 177.000, p=0.000, p<0.05).

When the correlations of ADMA and NP parameters with each other and with other parameters were examined, there was also a strong positive correlation between ADMA and NP values in obese individuals (r = 0.954). There was a positive correlation among ADMA/BMI (r=0.217), ADMA/HOMO-IR (r=0.276), and NP/BMI (r=0.476), NP/HOMO-IR (r=0.488).

With this study, we also aim to reveal the relationship between some cardiovascular risk factors such as diabetes, hypertension, some cardiovascular diseases and ADMA and NP parameters in obese individuals. A statistically significant difference was found between obese and healthy subjects in terms of the cardiovascular risk factors evaluated. (0.000<p<0.009). At the same time, there was a positive correlation between risk factors and test parameters (ADMA and NP) (0.033<r<0.736). There was a significant positive correlation between cardiovascular diseases and ADMA and NP (r=0.485, r=0.736, respectively).

## Discussion

In our study, ADMA and neopterin levels, their relationship with each other and with clinical and metabolic parameters and with cardiovascular risk factors were aimed to be investigated in obese and healthy subjects. The values of demographic and biochemical parameters were found to be significantly higher in obese individuals as compared to those in the controls (p<0.05). A positive correlation was found among all parameters (weight, BMI, Fasting Blood Glucose, Insulin, HOMA-IR, Total-Cholesterol, LDL-Cholesterol and Trglyceride. In addition, strong positive correlations were found between HOMA-IR and Fasting Blood Glucose, Insulin, Total-Cholesterol, LDL-Cholesterol and Triglyceride (0.365<r<0.964). The relationship between obesity and the changes in lipid metabolism is well known. In a study by Matsubara et al. [16], it was found that as body mass index increased, total cholesterol, triglyceride levels increased and HDL-c levels decreased. In another study conducted by Baskın et al. [17], it was found that TG levels increased and HDL-c levels decreased in obese people compared to the control group. No statistically significant relationship was found between obesity and Total cholesterol, LDL-c levels [17]. In another study by Şentürk et al. [18], serum total cholesterol, TG, LDL-c levels were found to be significantly higher in obese subjects compared to the control group. In a study by Shuldiner et al., insulin resistance was seen to have developed in all rat models where obesity was induced by the fructose diet. A high correlation was found between obesity and insulin resistance [19] in another study conducted by Baskin et al. [17], insulin and HOMA-IR levels were increased in obese subjects compared to the control group.

In our study, asymmetric dimethylarginine (ADMA) and neopterin (NP) biomarkers, which are important in cardiovascular diseases, were evaluated as obesity is a multifactorial, complex, and chronic disease and its prevalence has been rising at an alarming rate. ADMA is an endogenous nitric oxide which synthesizes (NOS) inhibitor that limits NO bioavailability [20]. Reduction of NO release causes elevated blood pressure and endothelial dysfunction develops. Endothelial dysfunction has been recognized to play a significant role in a number of circumstances associated with a high prevalence of atherosclerotic cardiovascular disease [15]. Impaired NO bioavailability and vascular dysfunction could result from an increase in DMA [7]. Previous studies reported that high ADMA plasma concentrations stand as a risk marker for a variety of cardiovascular diseases and with total mortality [9] [20] [21]. In our study, increased ADMA values were found in obese subjects as compared with the levels found in healthy subjects. Similarly, several previous studies found that plasma ADMA levels were elevated in obese [22] [23] and diabetic subjects compared to healthy controls [24] [25].

Obesity is related to endothelial dysfunction through direct mechanisms including proinflammatory adipokines production and elevated free fatty acids levels by adipose tissue, and also through indirect mechanisms such as insulin resistance and the related risk factors [26]. The involvement of mitochondrial superoxide and inflammation in the impairment of NO-mediated vasodilatation was determined by the presence of insulin resistance in human morbid obesity [27]. It was previously reported that increased ADMA levels contributed to the endothelial dysfunction as a result of the presence of insulin resistance in morbidly obese subjects [7]. McLaughlin et al. [28] demonstrated that obese but apparently healthy women who are insulin sensitive have lower plasma ADMA levels than equally obese but apparently healthy women who are insulin resistance. Erdogmus et al. (2009) found in patients with metabolic syndrome serum ADMA that Hcy and MDA levels were significantly higher than in the control group (p< 0.05) [29].

Obesity also presents as chronic, low-grade inflammation, and activation of the innate immune system [30]. Currently, the role of immune activation and inflammation in atherogenesis is well-established, and macrophages activation appears to be of crucial relevance within this process [31]. Researches have demonstrated an association between atherosclerosis and neopterin [32]. Neopterin is a noteworthy biomarker of systemic adaptive immune activation synthesized by macrophages activated by IFN-γ [31] [32]. It was previously reported that the levels of neopterin were significantly higher in obese adolescents with enhanced glucose levels for 30 min in comparison to those in both impaired glucose tolerance and normal glucose tolerance groups during oral glucose tolerance tests [33]. Agacayak et al. [34] reported that there was no significant difference between obese and non-obese patients with PCOS and control individuals in terms of neopterin. It was shown that serum neopterin levels did not show any significant difference between patients with and without obesity and obstructive sleep apnea; however, neopterin levels were positively correlated with BMI [32]. A previous study on non-obese and obese patients with obstructive sleep apnea syndrome reported that there was no significant difference between groups in terms of neopterin levels [35]. Besides, Yuniatry et al. [36] found that there was no correlation between neopterin and ADMA levels in metabolic syndrome patients. They concluded that the expression of these proteins might be independent of each other. However, in our study, enhanced neopterin levels were found in obese individuals with insulin resistance compared to controls. These findings seem to be associated with obesity-induced chronic inflammation. Another finding standing out in the present study is that high ADMA levels in obesity are associated with high neopterin levels. Taken together, our data suggested that endothelial dysfunction and inflammation were associated with obesity. In our study, the relationship between ADMA and NP and some biochemical parameters was investigated. There was a positive correlation between ADMA/BMI, ADMA/HOMO-IR, NP/BMI and NP/HOMO-IR. Similarly in a study, obese adolescents presented significantly higher triglycerides, cholesterol, glucose, insulin, HOMA-IR, and ADMA levels [37]. A strong relationship between BMI and plasma ADMA levels has also been reported to be a link to endothelial dysfunction in overweight individuals [38]. Moreover, it was revealed that serum ADMA levels were increased in postmenopausal obese women compared to their age-matched, normal-weight counterparts and that BMI was correlated positively with ADMA levels [39]. One consistent finding in most of these studies is that elevated ADMA levels are significantly associated with obesity. In the present study, it was shown that serum ADMA concentrations were increased in obese subjects with insulin resistance. These results suggested that the elevation of ADMA levels may play an important role in endothelial dysfunction associated with obesity and the presence of insulin resistance in these subjects may have an additive effect on ADMA levels. In addition, obesity-induced IR may be an additive effect on ADMA levels.

A statistically significant difference was found between obese and healthy subjects in terms of the risk factors evaluated in our study. There are many cardiovascular risk factors such as age, gender, family and history of cardiovascular disease among firstdegree relatives which are considered to be among unchangeable factors while smoking, hypertension, sedentary life, diabetes mellitus, dyslipidemia, obesity, left ventricular hypertrophy, microalbuminuria are defined as modifiable risk factors [40]. In our study, there was a positive correlation between cardiovascular risk factors (diabetes, hypertension, smoking, and cardiovascular diseases) and test parameters ADMA and NP. Similarly, in Framingam et al. [41] study, hypercholesterolemia, hypertension and smoking are the main risk factors.

In conclusion, our study revealed a rise in biochemical parameter values and elevation in ADMA and neopterin levels in obese subjects. Especially in our research, the relationship between the increased biochemical parameter levels and cardiovascular risk factors and ADMA and NP in obese individuals was demonstrated. Obesity leads to endothelial dysfunction and increases the risk of development of cardiovascular diseases. Therefore, understanding of the pathophysiology of obesity is very important for the early diagnosis of cardiovascular diseases in obesity. Our findings support the view that there is a relationship between endothelial dysfunction and systemic inflammation in obesity, and both ADMA and NP levels may be potential risk factors for cardiovascular disease in obesity.

## Conflict of interest statement

The authors declare that they have no conflicts of interest in this work.
